# Diagnostic value of cardiopulmonary ultrasound in elderly patients with acute respiratory distress syndrome

**DOI:** 10.1186/s12890-018-0666-9

**Published:** 2018-08-13

**Authors:** Daozheng Huang, Huan Ma, Zhiyuan Xiao, Michael Blaivas, Ying Chen, Jianyi Wen, Weixin Guo, Jun Liang, Xiaolong Liao, Zhonghua Wang, Hanbiao Li, Jie Li, Yangong Chao, Xiao ting Wang, Yan Wu, Tiehe Qin, Ke Su, Shouhong Wang, Ning Tan

**Affiliations:** 1 Department of Cardiology, Guangdong Cardiovascular Institute, Guangdong General Hospital and Guangdong Academy of Medical Sciences, Guangzhou, 510080 China; 2 Department of Critical Care Medicine, Yunnan Cancer Hospital and the Third Affiliated Hospital of Kunming Medical University, Kunming, 650000 China; 3 Department of Emergency Medicine, Piedmont Hospital, Newnan, GA USA; 4 Department of Critical Care Medicine, Guangdong Geriatric Institute, Guangdong General Hospital and Guangdong Academy of Medical Sciences, Guangzhou, 510080 China; 5 Department of Critical Care Medicine, the First Hospital of Tsing Hua University, Beijing, 100730 China; 6 Department of Critical Care Medicine, Peking Union Medical College Hospital, Peking Union Medical College, Chinese Academy of Medical Sciences, Beijing, 100730 China; 7 Department of Critical Care Medicine, Zhongshan Dongsheng Hospital, Zhongshan, 528400 China

**Keywords:** ARDS, Lung ultrasound, Cardiopulmonary ultrasound, Diagnostic value, Elderly

## Abstract

**Background:**

Lung ultrasound and echocardiography are mainly applied in critical care and emergency medicine. However, the diagnostic value of cardiopulmonary ultrasound in elderly patients with acute respiratory distress syndrome (ARDS) is still unclear.

**Methods:**

Consecutive patients admitted to ICU with the diagnosis of suspected ARDS based on clinical grounds were enrolled. Cardiopulmonary ultrasound was performed as part of monitoring on day 1, day 2 and day 3. On each day a bedside ultrasound was performed to examine the lungs and calculate the Left Ventricular Ejection Fraction (LVEF). On day 3, a thoracic CT was performed on each patient as gold standard for ARDS imaging diagnosis. According to the results from CT scan, patients were grouped into ARDS group or Non-ARDS group. The relation between the cardiopulmonary ultrasound results on each day and the results of CT scan was analyzed.

**Results:**

Fifty one consecutive patients aged from 73 to 97 years old were enrolled. Based on CT criteria, 33 patients were classified into the ARDS group, while 18 patients were included in non-ARDS group. There was no significant difference between the two groups in baseline characteristics, including gender, age, underlying disease, comorbidities, APACHE II score, SOFA score, and PaO2/FiO2 ratio (*P* > 0.05). Lung ultrasound (LUS) examination results were consistent with the CT scan results in diagnosis of pulmonary lesions. The Kappa values were 0.55, 0.74 and 0.82 on day 1, day 2 and day 3, respectively. The ROC analysis showed that the sensitivity, specificity and area under curve of ROC (AUROC) for lung ultrasound in diagnose ARDS were 0.788,0.778,0.783;0.909,0.833,0.871;0.970,0.833,0.902 on day 1, day 2 and day 3, respectively. However, cardiopulmonary ultrasound performed better in diagnosing ARDS in elderly patients. The sensitivity, specificity and AUROC were 0.879,0.889,0.924;0.939,0.889,0.961;and 0.970,0.833,0.956 on day 1, day 2 and day 3, respectively. The combined performances of cardiopulmonary ultrasound, N-terminal pro-brain natriuretic peptide (NT-proBNP), and PaO2/FiO2 ratio improved the specificity of the diagnosis of ARDS in elderly patients.

**Conclusions:**

LUS examination results were consistent with the CT scan results in diagnosis of pulmonary lesions. Cardiopulmonary ultrasound has a greater diagnostic accuracy in elderly patients with ARDS, compared with LUS alone**.** The combined performances of cardiopulmonary ultrasound, NT-proBNP, and PaO_2_/FiO_2_ increased the specificity of the diagnosis of ARDS in elderly patients.

## Background

Acute respiratory distress syndrome (ARDS) is a clinical syndrome, which is a type of acute diffuse, inflammatory lung injury, leading to increased pulmonary vascular permeability, increased lung weight, and loss of aerated lung tissue [1]. ARDS has a high incidence rate and caries mortality of nearly 50% for patients with severe ARDS [2]. At present, there are limited methods of improving the accuracy of ARDS diagnosis in patients [3–5]. Elderly patients generally have a higher number of comorbidities and poorer homeostatic capability. The diagnosis of ARDS in this patient population is even more challenging and needs improvement [2]. Therefore, early diagnosis and intervention are very important to improve the prognosis for elderly patients with ARDS. The diagnosis of ARDS at our institution is largely based on change noted on lung ultrasonography (LUS). The data collection of thoracic X-ray, especially bedside X-ray examination, can be affected by various factors. Hence we found it to not be valuable in the diagnosis of ARDS [6, 7]. Even with advantages over chest X-ray, thoracic CT has limited usage among critically ill patients, due to its high risk in patient transport, high cost, and risk of radiation exposure. Therefore, there are various underlying difficulties in early diagnosis of ARDS, especially imaging diagnosis.

LUS has drawn increasing attention among clinical physicians recently [8–12]. The guideline of international evidence-based recommendations for point-of-care lung ultrasound [13] aimed to improve and standardize the clinical application and scientific research of lung ultrasound. However, there has yet to be a good comparison between point-of-care ultrasound (PoCUS) and the imaging gold standard of CT diagnosis of ARDS. Persistent questions include: what ultrasound signs are best for the diagnosis diagnose ARDS? What are the sensitivity and specificity of PoCUS in diagnosing ARDS? What is the effect of the underlying diseases of the patients in diagnosis, especially those in lungs? In order to answer these questions, we performed a prospective observational study using dynamic ultrasound monitoring and other clinical indicators on 51 elderly patients with suspected ARDS, in order to investigate the diagnostic value of cardiopulmonary ultrasound in this patient group.

## Methods

### Subjects

Elderly patients with suspected ARDS based on clinical grounds, admitted to the department of Critical Care Medicine, Guangdong Geriatric Institute, Guangdong General Hospital and Guangdong Academy of Medical Sciences between January 2014 February 2016 [1]. Inclusion criteria: age is greater than or equal to 65 years old; risk factors of ARDS, including pneumonia, aspiration pneumonia, sepsis, septic shock, coma, multiple injuries, pancreatitis or large quantity of infusion of blood products; acute onset within 1 week or occurrence of severe acute respiratory system syndrome; Respiratory rate of more than 20 breaths per minute or presence of respiratory distress; PaO_2_/FiO_2_ < 300 mmHg; acute respiratory failure which cannot be explained by cardiac insufficiency or fluid overload [2]. Exclusion criteria: age < 65 years; end stage malignant neoplasms; no consent given or not able to have thoracic CT. Fifty-one patients were enrolled to this study, in which, fifteen patients with aspiration pneumonia, eleven patients with severe pneumonia, 10 patients with acute exacerbation of chronic obstructive pulmonary disease (AECOPD), eight patients with septic shock, seven patients with other diseases (including three cases with acute severe pancreatitis, two cases with multiple injury and two cases with cerebral hemorrhage). These included patients were divided into ARDS group (*n* = 33) and Non-ARDS group (*n* = 18), based on the results of thoracic CT scan on day 3. This study protocol was approved by the ethics committee of Guangdong General Hospital and Guangdong Academy of Medical Sciences [No. GDREC2015106H (R1)], and written informed consent was obtained from each patient’s next of kin.

### Instrument and diagnosis criteria

1.2.1 A Phillips EPIQ5 ultrasonic diagnostic apparatus was used. An abdominal convex array probe (Frequency 1-5 MHz) was used for the lung ultrasound examination; A phased array probe (frequency 1-5 MHz) was used for the cardiac ultrasound examination.

#### LUS examinations

**Fig. 1:**
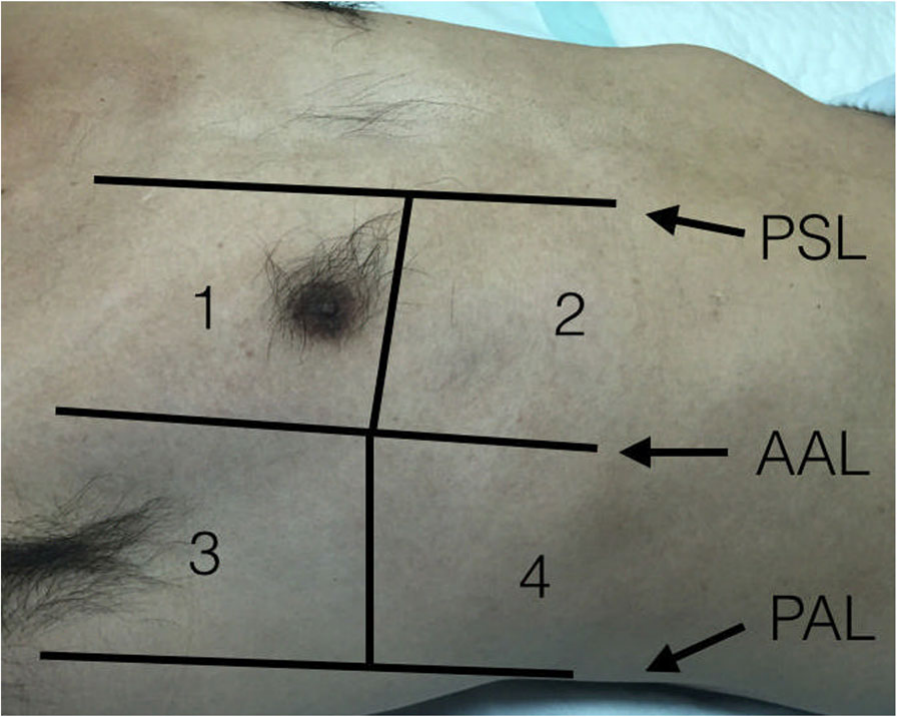
The four chest areas per side considered for complete twelve- zone lung ultrasound examination. Areas 1 and 2 denote the upper anterior and lower anterior chest areas, respectively. Areas 3 and 4 denote the upper lateral and basal lateral chest areas, respectively. PSL parasternal line, AAL anterior axillary line, PAL posterior axillary line.

**Fig. 2:**
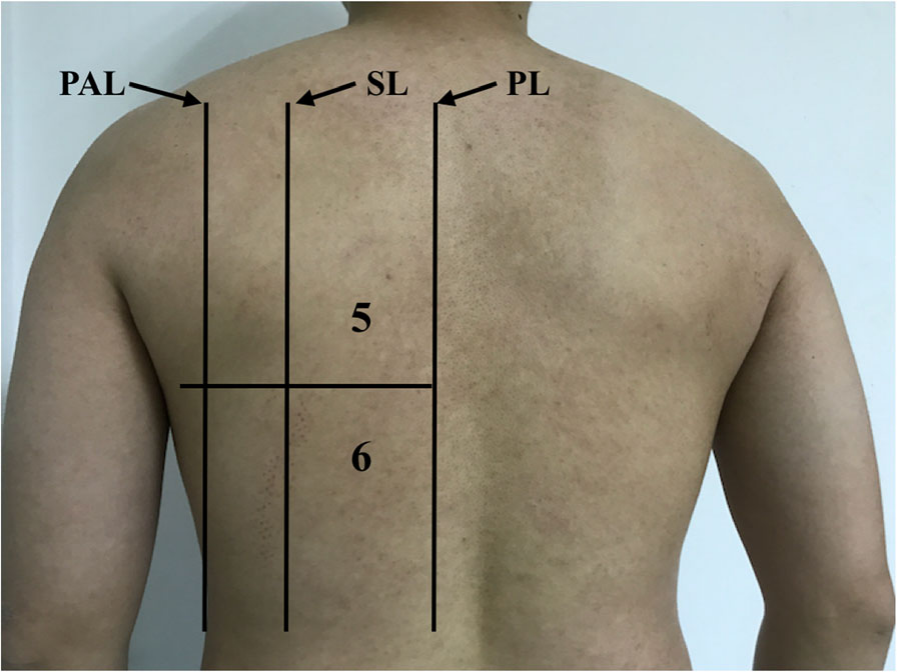
Areas 5 and 6 denote the upper backside and basal backside chest areas, respectively. PL paravertebral line, SL scapular line, PAL posterior axillary line

**Fig. 3:**
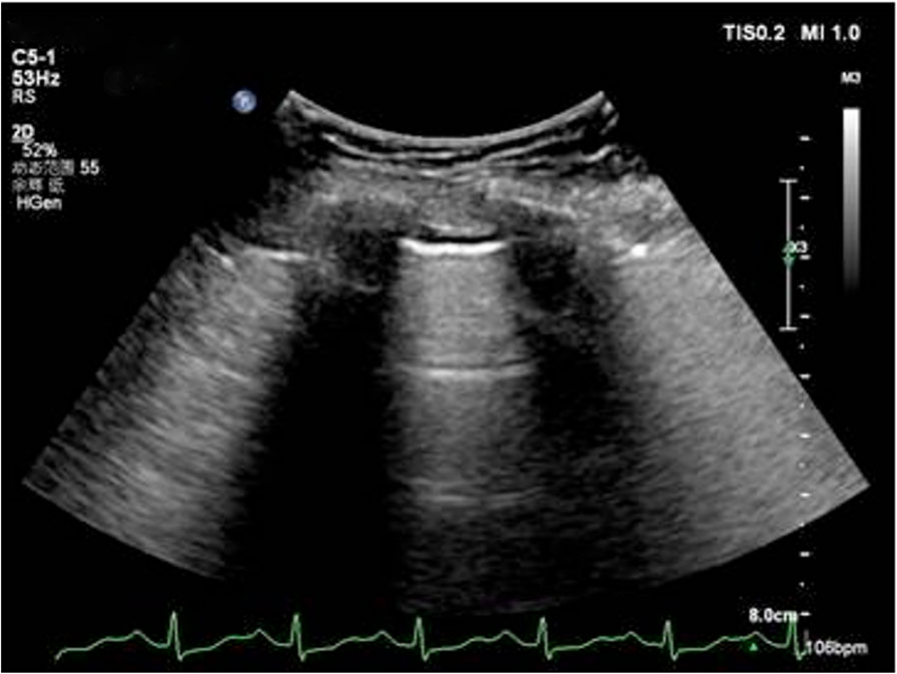
Sonographic signs of lung Normal pattern: sliding sign plus A-lines or less than three B-lines in a longitudinal plane between two ribs

**Fig. 4:**
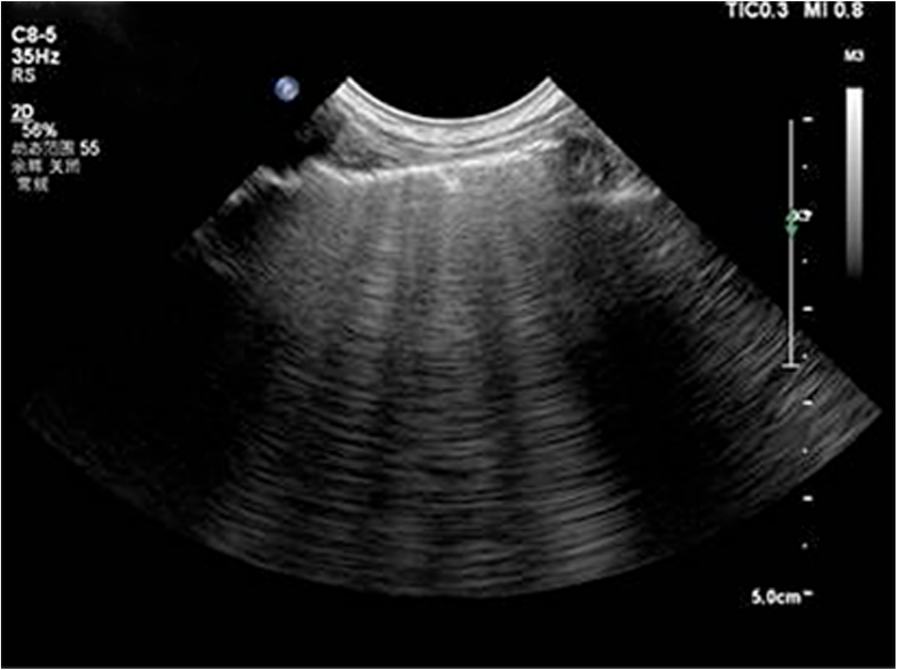
Interstitial syndrome: two or more positive regions bilaterally, A positive region is defined by the presence of three or more B-lines in a longitudinal plane between two ribs

**Fig. 5:**
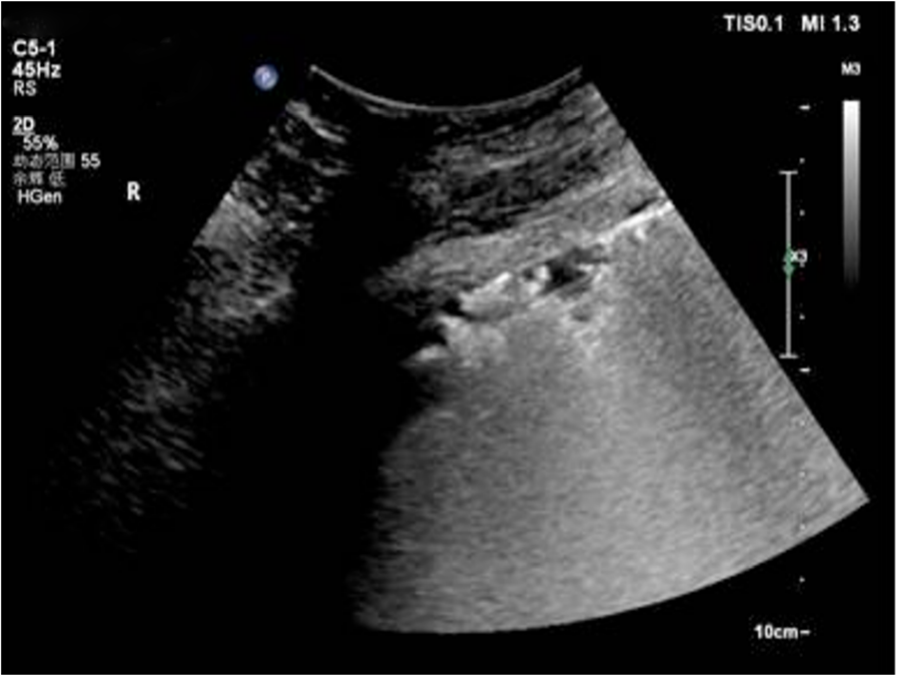
Shred sign:This consolidation is non-translobar and has the expected fractal, shredded border with the black aerated lung

**Fig. 6:**
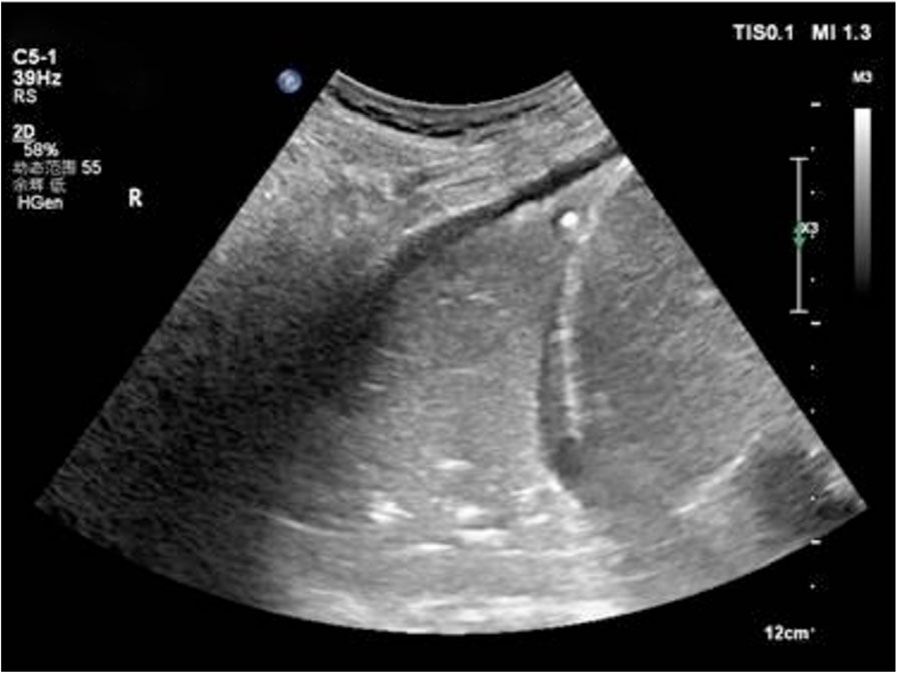
Lung consolidation: the sonographic sign of lung consolidation is a subpleural echo-poor region or one with tissue-like echotexture

**Fig. 7:**
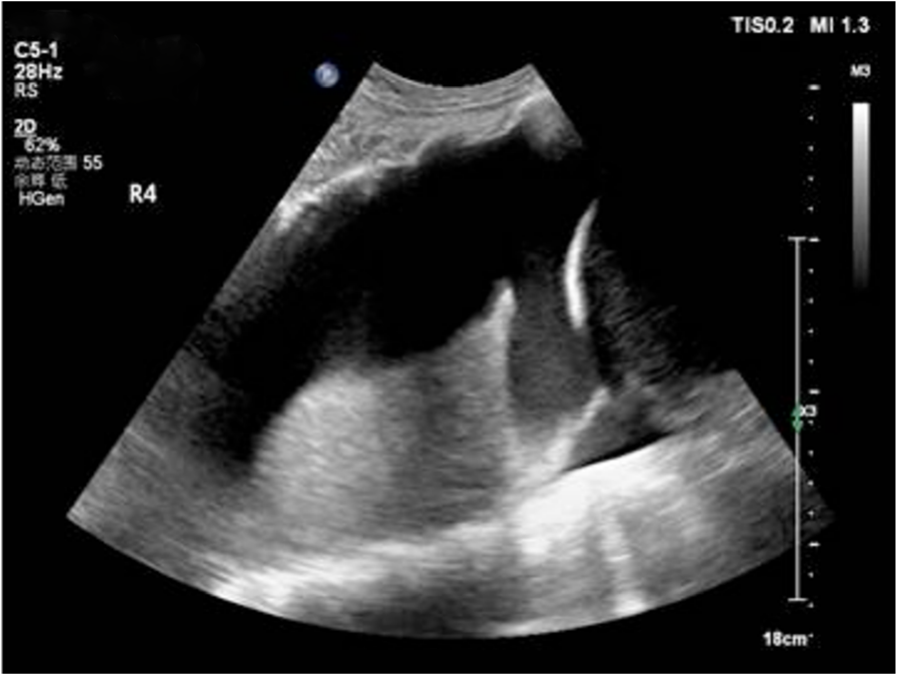
Pleural effusion: A space (usually anechoic) between the parietal and visceral pleura

**Fig. 8:**
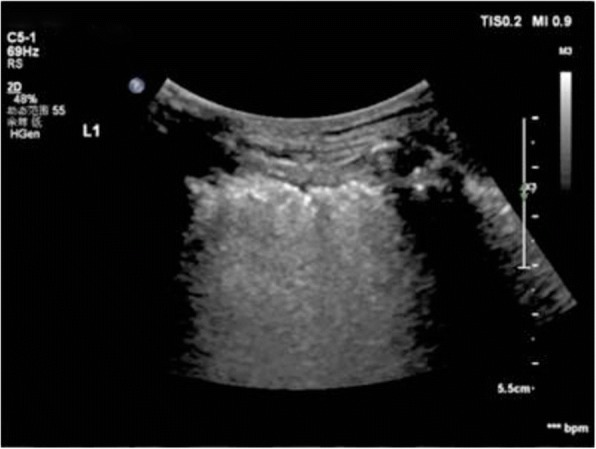
Pleural line abnormalities: irregular thickened fragmented pleural line

The examined locations were decided using a 12 partition method (Fig. 1). The ultrasound scan was operated using the longitudinal scan recommended by the international consensus on LUS published in 2012. The following ultrasound signs were set as indicative of an ARDS diagnosis (Fig. 2) ① Normal pattern (Fig. 3); ② Interstitial syndrome (Fig. 4); ③Consolidation (Figs. 5 and 6); ④Pleural effusion (Fig.7); ⑤Pleural line abnormalities (irregular thickened fragmented pleural line, absence or reduction of lung sliding) (Fig. 8). Currently, there are no consistent standards in diagnosing ARDS using lung ultrasound. However, Respiratory failure of patients without ARDS was mainly caused by cardiogenic factors, such as acute cardiogenic pulmonary edema. Whereas for patients with ARDS, severe hypoxemia caused by acute respiratory failure will affect heart function [14], and manifest as pulmonary edema and consolidation [15, 16]. In ARDS and non-ARDS, it appears that there will be pulmonary edema. How to differentiate between acute cardiogenic pulmonary edema in non-ARDS and ARDS? In contrast to cardiogenic pulmonary edema, the sonographic findings that are indicative of ARDS include the following: anterior subpleural consolidations, absence or reduction of lung sliding, “spared areas” of normal parenchyma, pleural line abnormalities (irregular thickened fragmented pleural line) and nonhomogeneous distribution of B-lines [13]. In this analysis,the ARDS diagnostic image standard include the following:that bilateral lung fields must have a combination of the two ultrasound signs of interstitial syndrome and consolidation plus other signs, such as normal pattern, pleural effusion and pleural line abnormalities. There were at least three or more signs required. The imaging data of all patients was prospectively documented and stored. These US evaluations were performed prospectively. The results of ultrasound examinations were analyzed blindly by two intensivists trained with critical ultrasonography. In case of a disagreement, the final decision was made after discussion between three experienced ultrasound clinical physicians with specific expertise in ARDS. All US evaluations were blinded from chest CT scan results.

#### Cardiac ultrasound

Cardiac ultrasound was evaluated using the standard sections, recommend by the American College of Cardiology Foundation (ACCF) 2011 [17]. Left and right heart function was evaluated by cardiac ultrasound utilizing an apical four chamber, parasternal long axis, parasternal short axis and subxiphoid views. LVEF and stroke volume (SV) were measured using an apical double plane Simpson method, in order to assess left ventricular systolic function. If sound transmission windows were inadequate, the LVEF was then estimated visually, to exclude left ventricular failure caused by acute respiratory failure. In the studied population, if LVEF≤35%, the patient with short of breath was caused by acute left heart failure, and these patients were classified as non-ARDS. Acute left heart failure was ruled out if LVEF≥50%. If LVEF is between 35 and 50%, the clinical symptomatology and lung ultrasound findings of the patients were considered to differential diagnosis of ARDS or non-ARDS. Right ventricular dysfunction (RVD) was defined using semi-quantitative size and function, fractional area change and tricuspid annular plane systolic excursion and tissue Doppler systolic velocity. In this study, we evaluated RVD through the shape of right ventricular by eyeballing. RVD was defined using end-diastolic RV/LV area ratio > 0.6. There was 91 % of patients had adequate US views for LVEF and SV to be measured, and five patients were excluded for poor US images. The cardiac US images were collected and analyzed by two investigators and the inter-observer variability was good.

#### Definition of ARDS

Bedsides onset time, risk factors and chest radiography, the Berlin definition proposed three mutually exclusive categories of ARDS according to PaO_2_/FIO_2_ ratios: mild (200 mmHg<PaO_2_/FIO_2_ ≤ 300 mmHg), moderate (100 mmHg<PaO_2_/FIO_2_ ≤ 200 mmHg), and severe (PaO_2_/ FIO_2_ ≤ 100 mmHg) and four ancillary variables for severe ARDS: radiographic severity, respiratory system compliance (≤40 mL/cm H O), positive end expiratory pressure (≥10 cm H_2_O), and corrected expired volume per minute (≥10 L/min) [1].

#### Thoracic CT scan

A Siemens 128-layer dual-source spiral CT (Germany) was used to perform the CT scan. Followed the implemented program, patients were supine, the layer distance was set as 5 mm. The scan duration was 2.5–3 min. Bilateral opacities consistent with pulmonary edema on the CT scan were considered as defining criteria for ARDS [1].

#### Definition of sepsis and septic shock

Sepsis is defined as the presence (probable or documented) of infection coupled with systemic manifestations of infection. Septic shock is defined as sepsis-induced hypotension persisting despite adequate fluid resuscitation, and vasopressors were needed to maintain mean atrial pressure (MAP) ≥ 65 mmHg [18].

#### Other procedures

The blood tests for NT-proBNP and arterial blood gas (ABG) analyses were drawn on the day of admission (labeled as day 1), day 2 and day 3, and test time of the 2 days thereafter were 7:30 a.m. The levels of NT-proBNP were measured by enzyme immunoassay. The upper limit of normal for apparently healthy persons (95th percentile) was 125 pg/mL for NT-proBNP. ABG blood samples for the measurement of ABG were obtained by arteriopuncture. ABG analyses were measured by bedside blood gas analyzer (ABL 800, RADIOMETER, Denmark). All operations are carried out in accordance with the operating procedures.

### Research methods

On day 1, day 2 and day 3, lung ultrasound was monitored and the LVEF values were measured for this elderly patient sample with acute respiratory failure, who met the inclusion criteria. A positive cardiopulmonary US exam for ARDS was defined as follow: [1] bilateral lung fields must have a combination of two ultrasound signs of interstitial syndrome and consolidation plus other signs [2]. LVEF≥50%. If LVEF is between 35 and 50%, the clinical symptomatology and lung ultrasound findings of the patients were considered to resolve the possibility of left heart failure [3]. If bilateral lung fields had less than three US finds or LVEF ≤35%, diagnosis of ARDS would be excluded. According to the results of CT scan, patients were stratified into ARDS group and non-ARDS group (control group). The relation between cardiopulmonary ultrasound monitoring results and the results of CT scan was analyzed.

### Statistics

Data were analyzed using a SPSS 19.0 statistic software. Metric data that were consistent with normal distribution were analyzed using $\overline{\mathrm{x}}\pm \mathrm{S}$, with a t-test comparison between two groups. Metri data that were not consistent with normal distribution were represented by average rank, with the Mann-Whitney rank sum test to compare the two groups. The comparison of the count data between groups was tested using Pearson *X*^*2*^_._ The ROC curve analysis was used for the second classification diagnosis. The results of different monitoring methods were tested for consistency. All *P* values were yielded from a bilateral test, if P<0.05, the difference was statistical significant.

## Results

### Comparison of the baseline characteristics of ARDS and control groups

Fifty one elderly patients with acute respiratory failure and suspected ARDS were enrolled, among which, 39 were male. Mean age was 82 [73, 97] years. 33 patients fell into the ARDS group, while 18 patients fell into the control group (non ARDS). There was no significant difference between the two groups in the baseline characteristics, including sex, age, underlying disease, APACHE II score, SOFA score, PaO_2_/FiO_2_ on day1, PEEP on day 1 and respiratory failure distance at screening, etc. On day 1, there was an increase in the NT-proBNP levels of the patients in both groups. NT-proBNP levels at admission were significantly higher in the control group compared with ARDS group (995.7 vs. 3229.0,Z = − 1.999,*p* = 0.046) (Table 1).

**Table 1:** Comparisons of the baseline characteristics between the ARDS group and the non-ARDS group

Baseline	ARDS group	Non-ARDS group	Test statistic	*P* value
Gender			χ2 = 0.026	0.871
male	25(76.0)	14(78.0)		
Female	8(24.0)	4(22.0)		
Age (years)	82.52 ± 5.56	82.83 ± 5.22	*t* = −0.199	0.843
Diagnosis				
Aspiration pneumonia	9	6	χ2 = 0.206	0.650
Severe pneumonia	7	4	χ2 = 0.007	0.933
AECOPD	7	3	χ2 = 0.153	0.696
Septic shock	5	3	χ2 = 0.020	0.877
Others	5	2	χ2 = 0.161	0.689
PaO_2_/FiO_2_ day1	140.45 ± 52.53	148.72 ± 35.40	*t* = −0.668	0.507
PEEP day1	8.27 ± 3.44	7.17 ± 3.00	*t* = 1.147	0.257
NT-proBNP day1	995.7 (339.4,5944.0)	3229.0 (838.6,16,339.0)	*Z* = −1.999	0.046^*^
APACHEIIscore	26.33 ± 5.69	25.44 ± 5.20	*t* = 0.549	0.585
SOFA score	9.48 ± 3.36	9.72 ± 2.95	*t* = −0.251	0.803
Time of respiratory failure(hours)	17.45 ± 18.97	15.83 ± 13.29	*t* = 0.321	0.749

day1 refers to the first day included in the study; respiratory failure time: refers to the patients with respiratory failure (with arterial blood gas as the diagnostic criteria, if not to check for blood gas analysis, clinical judgment as to the diagnostic criteria) included in the study of the time difference, in hours (H) said. ^*^The difference was statistically significant (*P* < 0.05)

2.2 LUS diagnostic results were consistent with CT results in both patient groups, based on the previously mentioned diagnostic standards of ARDS. The consistency test indicated that pulmonary ultrasound results were consistent with the CT results of the patients on day 1, day 2 and day 3, with Kappa values of 0.55, 0.74 and 0.82, respectively. The consistency of lung ultrasound and CT results was the highest on day 3 (Tables 2 and 3). All patients met criteria for ARDS on admission, and they continued to meet the Berlin criteria for ARDS on day 2 and day 3.

**Table 2:** Comparison of lung ultrasound and CT scan in the diagnosis of lung lesions in ARDS group and Non-ARDS group

Group	Method	No. of subjects	Normal pattern	Interstitialsyndrome	Lung consolidation	Pleural line abnormalities	Pleural effusion
ARDS group	CT	33	30	30	33	–	31
	Ultrasound						
	day1	30	29	32	32	17	27
	day2	33	28	32	33	16	28
	day3	35	29	32	33	17	32
non-ARDS group	CT	18	13	13	5	–	15
	Ultrasound						
	day1	21	15	16	3	1	10
	day2	18	12	14	3	1	11
	day3	16	16	13	3	1	14

**Table 3:** Consistency test of dynamic monitoring of LUS and CT scan

Time	ARDS group	Non-ARDS group	Kappa
LUS day1			0.55
+	26	4	
–	7	14	
LUS day2			0.74
+	30	3	
–	3	15	
LUS day3			0.82
+	32	3	
–	1	15	

2.3 The ROC analysis showed that the sensitivity, specificity and AUROC of using lung ultrasound to diagnose ARDS were 0.788,0.778,0.783;0.909,0.833,0.871;0.970,0.833,0.902 on day 1, day 2 and day 3, respectively (Table 4).

**Table 4:** Analysis of ROC curve of pulmonary dynamic monitoring results in patients with ARDS

LUS	Cutoff values	Sensitivity	Specificity	Youden index	AUROC
day1	1.0	0.788	0.778	0.566	0.783
day2	1.0	0.909	0.833	0.742	0.871
day3	1.0	0.970	0.833	0.803	0.902

### ROC curve analysis of cardiopulmonary ultrasound in diagnosing ARDS

The sensitivity, specificity and AUROC were 0.879,0.889,0.924;0.939,0.889,0.961;and 0.970,0.833,0.956 on day 1, day 2 and day 3, respectively (Table 5).

**Table 5:** The analysis of ROC curve in diagnosis of ARDS by dynamic monitoring of Cardiopulmonary ultrasound

Cardiopulmonary ultrasound	Cutoff values	Sensitivity	Specificity	Youden index	AUROC
day1	0.531	0.879	0.889	0.768	0.924
day2	0.499	0.939	0.889	0.828	0.961
day3	0.441	0.970	0.833	0.803	0.956

### ROC curve analysis of the combined performances of cardiopulmonary ultrasound and NT-proBNP in diagnosing ARDS

Combination of cardiopulmonary ultrasound and NT-proBNP or PaO_2_/FiO_2_ improved the specificity of the diagnosis of ARDS. AUROC values were > 0.90 on day 1, day 2 and day 3. The combination of LUS + LVEF+NT-proBNP had the highest diagnostic value on day 2, with sensitivity, specificity and AUROC of 0.939, 0.889 and 0.965, respectively (Table 6).

**Table 6:** ROC curve analysis of ARDS diagnosis with cardiopulmonary ultrasound combined with NT-proBNP or PaO_2_/FiO_2_ ratio

Combination Index	Cutoff Value	Sensitivity	Specificity	Youden index	AUROC
Cardiopulmonary ultrasound +NT-proBNP day1	0.556	0.879	0.889	0.768	0.938
Cardiopulmonary ultrasound +NT-proBNP day2	0.487	0.939	0.889	0.828	0.965
Cardiopulmonary ultrasound +NT-proBNP day3	0.745	0.939	0.889	0.828	0.961
Cardiopulmonary ultrasound + PaO_2_/FiO_2_ day1	0.764	0.848	0.944	0.793	0.919
Cardiopulmonary ultrasound + PaO_2_/FiO_2_ day2	0.496	0.939	0.889	0.828	0.962
Cardiopulmonary ultrasound + PaO_2_/FiO_2_ day3	0.442	0.970	0.833	0.803	0.958

2.6 ROC curve analysis of cardiopulmonary ultrasound+NT-proBNP+ PaO_2_/FiO_2_ diagnosing ARDS: The above combination was of high value with AUROC> 0.920 on day 1, day 2 and day 3; In which, the diagnostic value was the highest on day 2, with sensitivity, specificity and AUROC of 0.939,0.889 and 0.965, respectively (Table 7).

**Table 7:** The ROC curse analysis of Cardiopulmonary Ultrasound combined with NT-proBNP and PaO_2_/FiO_2_ ratio in the diagnosis of ARDS

Time	Cut-off value	Sensitivity	Specificity	Youden index	AUROC
day1	0.726	0.848	0.944	0.793	0.928
day2	0.486	0.939	0.889	0.828	0.965
day3	0.738	0.939	0.889	0.828	0.961

## Discussions

Chinese society is aging [19],and resulting in an increasing number of critically ill elderly patients. The mortality and morbidity of those patients may be much higher than younger patients. This may be attributed to a lower reserve capacity in most important organs and systems functions, which will reduce ability to deal with physical stress and the presence of acute or chronic comorbidities. Therefore, early diagnosis maybe have a marked impact on interventions and outcomes of elderly patients with ARDS. However, the Berlin diagnostic criteria of ARDS widely used in clinical practice, is not very clear about the evaluation standard of pulmonary imaging, especially thoracic X-ray, which may lead to poor reliability of ARDS diagnosis [8]. The development of clinical application and research of PoCUS provides a novel way of ARDS diagnosis in imagine. Cardiopulmonary ultrasound can help evaluate the cardiopulmonary morphology and function, but more exploration is needed to investigate the relation between cardiopulmonary ultrasound and chest CT scan in chest imagines for diagnosis of ARDS. If ultrasound proved sensitive and specific in early ARDS diagnosis, it may become part of a novel diagnostic imaging standard of ARDS diagnosis.

This study was a single-center, prospective observational study. Patients were divided into ARDS group (*n* = 33) and control group (*n* = 18), based on the results of chest CT scan on day 3. There were no significant differences between the two groups in gender, age, underlying disease, APACHEII score, SOFA score, PaO_2_/FiO_2_ ratio and PEEP levels day 1. It’s reported that NT-proBNP is an important biomarker of heart failure [20]. Previous studies suggested that patients with diagnosis of ARDS often had right ventricular dysfunction [21, 22], which would damage cardiopulmonary function. This study showed that, the levels of NT-proBNP on day 1 were increased in both groups, while the levels in control group were statistically significantly higher than those in the ARDS group (*p* = 0.046). Respiratory failure of patients without ARDS was mainly caused by cardiogenic factors, whereas for patients with ARDS, other factors, such as hypoxia, can also affect the cardiac function. There are obvious limitations to using NT-proBNP alone to differentiate ARDS from acute or chronic heart failure.

Our study showed that the results of LUS examinations were comparable with those of ARDS diagnosis using CT scan. Consistent with the results of the preliminary animal experiment in the research group, the Kappa value was 0.82 on day 3,indicating early ARDS diagnosis using LUS was of similar clinical value as a CT scan. In our study, the results of CT scan in 33 patients with ARDS on day 3 showed different degrees of pulmonary consolidation, especially obvious in the gravity-dependent areas, such as lateral chest and back. This is a characteristic finding of ARDS on chest CT scan. The relation between ultrasound signs and ARDS diagnostic criteria through chest CT scan was analyzed according to the ARDS diagnosis standard using ultrasound suggested in this study. The sensitivity, specificity and AUROC of LUS on day 1, day 2 and day 3 were high and increased between day 1 and day 3. Our results indicated that diagnostic imaging of ARDS could be partially based on LUS signs. The results on day 3 were of higher diagnostic value, since the third day may be the peak of the inflammation.

Pulmonary ultrasound yields considerable advantage in the diagnosis and management of various pleural cavity and pulmonary diseases [23–26]. Change in pulmonary ventilation area can be determined by LUS before the reduction of PaO_2_/FiO_2_ [27]. Our previous animal experiment showed that LUS could semi-quantify pulmonary edema [28, 29]. Inevitably, the diagnosis of ARDS must be differentiated from acute left heart failure, and cardiac ultrasound is an important means of comprehensive evaluation of cardiac function. Previous studies showed that cardiopulmonary ultrasound had obvious advantages in investigating the etiology of acute respiratory failure [30, 31]. Therefore, we believe that a cardiopulmonary ultrasound approach will have broad application prospects in ARDS diagnosis. The sensitivity, specificity and AUROC of ARDS diagnosis using cardiopulmonary ultrasound on day 1, day 2 and day 3 were 0.879,0.889,0.924;0.939,0.889,0.961;and 0.970,0.833,0.956, respectively.

Our study’s main aim was to investigate the diagnostic value of cardiopulmonary ultrasound in elderly patients with ARDS. According to the guideline of point of care LUS [13], there are no consistent standards in diagnosing ARDS using LUS findings. Combined with Berlin criteria of ARDS [1] and our clinical experience, pulmonary edema and consolidation are the main imaging findings in ARDS patients. If there is normal LVEF or even hyperdynamic state in patients, combined with the results of LUS,we basically believe that the patient’s respiratory distress is caused by pulmonary edema caused by ARDS. Our results showed that cardiopulmonary ultrasound was of greater advantage compared with LUS alone in ARDS diagnosis. The reason may be that cardiopulmonary ultrasound can exclude the interference of heart failure in ARDS diagnosis. Both NT-proBNP and PaO_2_/FiO_2_ are important clinical indicators in pathophysiology. We also evaluated the combination of cardiopulmonary ultrasound and NT-proBNP or PaO_2_/FiO_2_ in ARDS diagnosis, with sensitivity, specificity and AUROC on day 2:0.938,0.887,0.964;0.939,0.889,0.965, respectively. These results suggest a combination of ultrasound signs and pathophysiology indicators was of greater application value in ARDS diagnosis.

This study investigated the ARDS diagnosis in elderly patients using PoCUS. Lung ultrasound, especially cardiopulmonary ultrasound was of important clinical application value in ARDS diagnosis in elderly patients. A combination of ultrasound signs and pathophysiology indicators was of more application value than ultrasound signs alone. PoCUS is mainly applied not only in critical care, emergency medicine, and trauma surgery, but also in pulmonary and internal medicine, especially in the assessment of cardiopulmonary function. Respiratory failure of patients without ARDS was mainly caused by cardiogenic factors, whereas for patients with ARDS may be promoted in the diagnosis and treatment management in elderly patients with ARDS.

This study had a few of limitations including its observational design and finite study arms. Initial indications are promising for cardiopulmonary ultrasound in the diagnosis of ARDS. However, the effect of underlying disease of elderly patients on its diagnostic value needs to be investigated in larger prospective and also interventional studies to evaluate any effect on outcomes.

## Conclusions

Cardiopulmonary ultrasound has a greater diagnostic accuracy in elderly patients with ARDS, compared with lung ultrasound alone. The combined performances of cardiopulmonary ultrasound, NT-proBNP, and PaO_2_/FiO_2_ increased the specificity of the diagnosis of ARDS in elderly patients.

## Abbreviations

ACCF: American College of Cardiology Foundation
AECOPD: Acute exacerbation of chronic obstructive pulmonary disease
ARDS: Acute respiratory distress syndrome
AUROC: Area under the ROC
LUS: Lung ultrasound
LVEF: Left ventricular ejection fraction
NT-proBNP: N-terminal pro-brain natriuretic peptide
PoCUS: Point-of-care ultrasound
ROC: Receiver operating characteristic curve
SV: Stroke volume
US: Ultrasonography
